# Heartbreak From Gilteritinib: Two Case Reports of Delayed Onset Cardiotoxicity

**DOI:** 10.1155/cric/1976122

**Published:** 2025-04-29

**Authors:** Kristi Dutta, Ethan D. Kotloff, Manu M. Mysore

**Affiliations:** ^1^Department of Medicine, University of Maryland School of Medicine, Baltimore, Maryland, USA; ^2^Division of Cardiovascular Medicine, University of Maryland School of Medicine, Baltimore, Maryland, USA

**Keywords:** cardio-oncology, gilteritinib, heart failure

## Abstract

Activating mutations of FMS-like Tyrosine Kinase 3 (FLT3) occur in a subset of patients with acute myeloid leukemia (AML) and confer a poor prognosis. Gilteritinib, an oral FLT3 inhibitor approved for the treatment of relapsed or refractory AML, has been shown to improve survival and remission rates compared with salvage chemotherapy. This case report presents two patients initiated on gilteritinib for relapsed AML who developed new onset left ventricular systolic dysfunction. After ruling out other common etiologies, gilteritinib was discontinued due to concern for cancer therapy–related cardiac dysfunction with subsequent improvement in ejection fraction. These cases demonstrate a rare but serious adverse effect of gilteritinib, cardiotoxicity manifested as left ventricular systolic dysfunction, for which more studies are needed to elucidate the underlying pathophysiology.

## 1. Introduction

Mutations in FMS-like Tyrosine Kinase 3 (FLT3) occur in approximately 30% of patients with acute myeloid leukemia (AML) and confer a poor prognosis. Gilteritinib, an oral FLT3 tyrosine kinase inhibitor (TKI), was approved in 2018 for the treatment of relapsed or refractory AML after it was demonstrated to improve survival and remission rates compared with salvage chemotherapy [1]. While gilteritinib and other TKIs are generally less toxic than conventional chemotherapy, there is evidence that these medications may be associated with unforeseen, adverse cardiovascular events [2]. Here, we present two cases of gilteritinib-associated cardiomyopathy in patients with relapsed AML.

## 2. Case 1

### 2.1. Presentation and Physical Examination

A 67-year-old man with relapsed FLT3-mutated AML on gilteritinib presented to clinic with a complaint of 2 weeks of intermittent left-sided chest pressure exacerbated by stress. He endorsed only mild dyspnea on exertion. Systolic blood pressure was elevated, but other vital signs were otherwise within normal limits, and there were no pertinent findings on physical exam.

**Figure 1:**
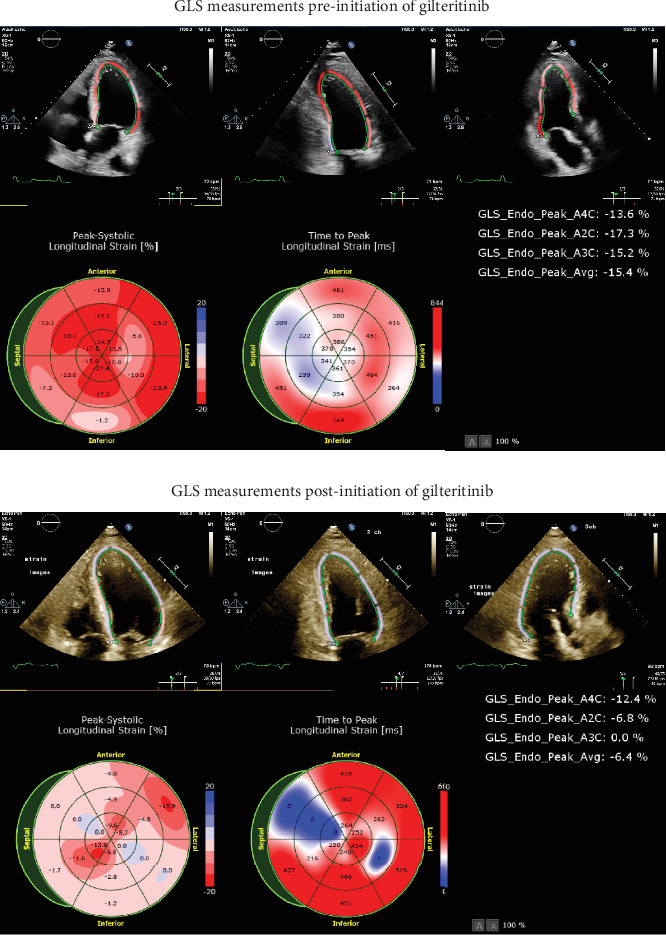
Global longitudinal strain (GLS) measurements pre- and postinitiation of gilteritinib in Case 1.

Laboratory evaluation revealed Troponin I of 0.03 ng/mL and *N*-terminal pro b-type natriuretic peptide (NT-proBNP) of 102 pg/mL. Electrocardiogram (ECG) showed normal sinus rhythm with left bundle branch block, which was unchanged from prior. Transthoracic echocardiogram (TTE) demonstrated newly reduced left ventricular ejection fraction (LVEF) of 30%–35% with global longitudinal strain (GLS) of −6.4% from a normal TTE with LVEF of 55% and GLS of −15.4% 6 months prior, just before initiation of gilteritinib (Figure 1).

### 2.2. Medical History

Past medical history included obesity, hypertension, hyperlipidemia, type 2 diabetes mellitus, tobacco use, chronic kidney disease (CKD), and coronary artery disease (CAD) with placement of one drug-eluting stent to the left anterior descending (LAD) artery. Family history was significant for myocardial infarction (MI) in his mother and two siblings. He was initially diagnosed with AML 2 years before this presentation and had completed 7 + 3 induction chemotherapy with cytarabine and idarubicin followed by consolidation therapy with high-dose cytarabine. The patient had a normal TTE prior to induction therapy. He relapsed with high-risk MDS (FLT3-ITD mutation) for which he was started on gilteritinib 120 mg daily. He was maintained on this dose throughout the course of therapy—in total 8 months.

### 2.3. Differential Diagnosis

The differential diagnosis included ischemic cardiomyopathy, hypertensive heart disease, myocarditis, and cancer therapy–related cardiac dysfunction (CTRCD). Pulmonary causes of chest discomfort including pulmonary embolism, pulmonary hypertension, and pulmonary malignancy were also considered.

### 2.4. Management

The patient was referred for coronary angiography, which revealed nonobstructive CAD and a patent LAD stent. He was later initiated on lisinopril and carvedilol as part of guideline-directed medical therapy (GDMT). After deliberation between his oncologist and cardiologist, gilteritinib was discontinued due to concern for CTRCD. Gilteritinib was briefly restarted 2 months later in the setting of peripheral blasts consistent with relapsed AML and persistent FLT3 mutation, with the understanding that cardiac function may continue to decline. However, therapy was discontinued 1 month later due to concern for gilteritinib resistance.

### 2.5. Outcome

A repeat TTE performed 3 months after discontinuation of gilteritinib showed improvement in LVEF to 40%. He remained free of signs and symptoms of heart failure.

### 2.6. Latest Follow-Up

The patient underwent four cycles of azacitidine and venetoclax without having achieved complete remission.

## 3. Case 2

### 3.1. Presentation and Physical Examination

A 76-year-old female with relapsed FLT3/IDH2 mutated AML on gilteritinib and enasidenib presented to the emergency department with several weeks of progressive weakness, fatigue, lightheadedness, dyspnea on exertion, and lower extremity edema. She denied chest pain, palpitations, or syncope. Her blood pressure was 111/63 mmHg supine but decreased to 84/53 standing. She was mildly tachycardic but otherwise afebrile with normal oxygen saturation on room air. Physical examination was notable for obesity, pitting edema of the bilateral ankles, and borderline elevated jugular venous pulsation measured at 9 cmH_2_O.

Laboratory workup revealed creatinine of 1.9 mg/dL compared to a baseline of 1.2, NT-proBNP of 498 pg/L, ferritin of 24,729 ng/mL, transferrin saturation of 98%, and thyroid-stimulating hormone (TSH) of 39.6 mIU/L. ECG showed sinus tachycardia without T-wave or ST-segment abnormalities but a prolonged QTc of 507 ms. TTE was significant for a newly reduced LVEF of 40%–45% with global hypokinesis and mild to moderate regurgitation noted in all four valves. Baseline TTE prior to induction therapy and TTE at the time of dose increase of gilteritinib to 200 mg daily (3 months prior) had an LVEF of 60%–65% and were without significant valvular abnormality.

### 3.2. Medical History

Past medical history included hypertension, hyperlipidemia, CKD, tobacco use, blood transfusions, and multinodular goiter status post thyroidectomy complicated by hypothyroidism. She had been diagnosed with FTL3/IDH2-mutated AML 1.5 years before presentation for which she underwent induction chemotherapy with aspacytarabine (BST-236) resulting in complete remission. The patient had a normal TTE prior to induction therapy. A repeat bone marrow biopsy performed 3 months later showed evidence of relapsed disease at which time she was initiated on gilteritinib and enasidenib. The patient received gilteritinib therapy for 2.5 years in total, initiated at a dose of 120 mg daily up to 200 mg daily after 1 year (3 months prior to the above presentation) due to persistent peripheral blasts, and was maintained on that dose. Enasidenib was maintained at 100 mg daily.

### 3.3. Differential Diagnosis

The differential diagnosis included ischemic cardiomyopathy, iron overload cardiomyopathy, hypothyroidism-related cardiac dysfunction, and CTRCD.

### 3.4. Management

During hospitalization, she received intravenous fluids for orthostatic hypotension and acute kidney injury and was restarted on levothyroxine for hypothyroidism. Prior to discharge, she was initiated on lisinopril as part of GDMT for heart failure. While CTRCD was on the differential for her cardiomyopathy and prolonged QTc, gilteritinib was continued after risk–benefit analysis.

Following discharge, the patient was seen in a cardio-oncology clinic where she was initiated on metoprolol succinate and empagliflozin per GDMT. She underwent a myocardial perfusion positron emission tomography (PET) stress test, which showed no evidence of ischemia or infarction but was suggestive of microvascular dysfunction with an LVEF of 50% at rest that improved to 56% with pharmacological vasodilation. Cardiac magnetic resonance imaging (CMR) showed no evidence of myocardial iron overload but was notable for a small pericardial effusion as well as edema and inflammation in the basal to midinterventricular septum suggestive of an inflammatory or infiltrative process. The patient remained on gilteritinib for six more months until it was discontinued due to platelet dysfunction, with repeat bone marrow biopsy negative for FTL3 mutation. Patient was continued on enasidenib.

### 3.5. Outcome

A repeat TTE performed 1 month after discontinuation of gilteritinib showed improvement in LVEF to 55%–60% with a trivial pericardial effusion.

### 3.6. Latest Follow-Up

Due to rising blast count, enasidenib was stopped. The patient underwent one cycle of azacytidine and venetoclax for treatment of relapsed AML. Shortly after discharge, she was hospitalized for neutropenic fever and ultimately opted for home hospice.

## 4. Discussion

These cases demonstrate the development of left ventricular systolic dysfunction after gilteritinib therapy in two patients with relapsed AML. The patient in Case 1 had risk factors for ischemic cardiomyopathy including obesity, hypertension, dyslipidemia, diabetes, tobacco use, CAD, and CKD, but coronary angiogram showed no significant stenoses and a patent LAD stent. He had no evidence of hypertrophy on ECG or TTE to suggest hypertensive cardiomyopathy, no history of tachyarrhythmia to suggest arrhythmia-induced cardiomyopathy, no valvular abnormalities on TTE, and no evidence of pulmonary causes on imaging or TTE. Given the exclusion of other etiologies in addition to the improvement in LVEF following discontinuation of gilteritinib, the most likely cause of cardiomyopathy was gilteritinib-associated cardiotoxicity with a score of 6 (probable) on the Naranjo Adverse Drug Reaction Probability Scale [3].

The patient in Case 2 also had risk factors for ischemic heart disease, but a PET stress test was negative for ischemia or infarction. Given her history of blood transfusions and markedly elevated ferritin and transferrin saturation, myocardial iron overload was on the differential, but CMR showed no evidence to support this diagnosis. Hypothyroidism was also considered a possible etiology; however, at the time of her repeat TTE documenting recovered LVEF, her TSH remained elevated, ruling this out. Although the patient was receiving enasidenib concurrently with gilteritinib, it was continued for 1 month after gilteritinib discontinuation, during which time a follow-up TTE showed improvement in cardiac function. The most likely cause of her cardiomyopathy, therefore, was gilteritinib-associated cardiotoxicity with a score of 7 (probable) on the Naranjo Scale [3]. In this patient case, the dose may be an important factor in the development of cardiomyopathy, given she had tolerated gilteritinib for a year and 3 months after dose increase had the above changes on TTE.

While rare, these are not the first cases of cardiotoxicity attributed to gilteritinib reported in the literature. Kim et al. described a similar case of new onset heart failure with evidence of myocarditis in a patient on gilteritinib for relapsed AML. After four doses of gilteritinib, the patient presented with dyspnea, orthopnea, lower extremity swelling, and weight gain and was found to have a newly reduced LVEF of 35%–40% from a normal baseline. Coronary angiogram was deemed too high risk due to thrombocytopenia. However, CMR demonstrated myocardial edema and inflammation in the inferoseptum consistent with myocarditis. Following discontinuation of gilteritinib, repeat CMR performed 5 months later revealed normalization of LVEF and resolution of myocardial inflammation [4]. Case 1 differs in that the patient was free from symptoms of volume overload or decompensation, suggesting that gilteritinib-associated cardiomyopathy may present on a spectrum from asymptomatic left ventricular dysfunction to overt heart failure. While CMR was not performed in Case 1, CMR in Case 2 showed a similar distribution of myocardial edema and inflammation in the basal septum, suggesting that gilteritinib may induce an inflammatory cardiomyopathy.

There is also evidence from clinical trials that gilteritinib has associated cardiotoxicity. In a Phase 1/2 open-label, dose-escalation study investigating the safety and tolerability of gilteritinib, 4% of patients experienced new onset heart failure. Additionally, 7% of patients had an increase from baseline QTc greater than 60 ms, 3% developed pericardial effusion, and 2% developed pericarditis [5]. In the ADMIRAL trial, a Phase 3 randomized controlled trial that established gilteritinib as the standard of care for relapsed or refractory AML, 5% of patients had QTc prolongation greater than 60 ms from baseline. While a significant proportion of patients reported nonspecific symptoms including fatigue (28.5%), cough (29.3%), peripheral edema (24.0%), and dyspnea (23.6%), other cardiovascular adverse events including heart failure, pericardial effusion, or pericarditis were not formally evaluated or reported [1].

The pathophysiology underlying gilteritinib-associated cardiotoxicity is unclear, and studies exploring the role of FLT3 signaling in the heart are sparse. Pfister et al. provided a possible mechanistic explanation using a mouse model of MI. After inducing MI in mice via ligation of the LAD, FLT3 ligand (FL) or vehicle was injected into the infarct border zone. Myocardial remodeling and function were assessed via echocardiography and histological analysis at baseline and 1 week following infarction. Furthermore, FLT3 expression and molecular mechanisms of FLT3 action were examined in vitro. The primary findings were (1) FL improved post-MI remodeling and function, (2) FLT3 expression by cardiomyocytes was upregulated in response to oxidative stress, and (3) FLT3 stimulation by FL induced prosurvival pathways in cardiomyocytes. Overall, the study provided evidence that FLT3 has an intrinsic cardioprotective role. The authors proposed that FLT3-targeting TKIs exert on-target cardiotoxic effects by suppressing FLT3-mediated antiapoptotic signaling pathways, resulting in increased cardiomyocyte death [6].

## 5. Conclusion

These cases demonstrate a seemingly rare but serious complication of gilteritinib: cardiotoxicity manifested as left ventricular systolic dysfunction. More studies are needed to understand the mechanism underlying gilteritinib-associated cardiotoxicity in humans as well as any dose-dependent injury to myocardial tissue. Furthermore, risk factors that predispose patients to gilteritinib-associated cardiotoxicity are unclear and warrant further study to help risk-stratify patients prior to the initiation of treatment. Lastly, protocols for monitoring of cardiovascular adverse events while on gilteritinib with clear indications for dose reduction or discontinuation of therapy should be developed. Consultation with a cardio-oncologist before and during treatment with gilteritinib may be beneficial to help mitigate the risk of cardiotoxicity, monitor for the development of cardiotoxic adverse events, and treat those that may arise.

## Data Availability

Data sharing is not applicable to this article as no datasets were generated or analyzed during the current study.
